# The German National Cohort: aims, study design and organization

**DOI:** 10.1007/s10654-014-9890-7

**Published:** 2014-05-20

**Authors:** German National Cohort (GNC) Consortium

**Affiliations:** Central Executive Office of the German National Cohort, Im Neuenheimer Feld 581, 69120 Heidelberg, Germany

**Keywords:** Population-based cohort, Non-communicable diseases, Chronic infections, Life-style and socio-economic factors, Magnetic resonance imaging, Pre-clinical disease, Functional impairments

## Abstract

The German National Cohort (GNC) is a joint interdisciplinary endeavour of scientists from the Helmholtz and the Leibniz Association, universities, and other research institutes. Its aim is to investigate the causes for the development of major chronic diseases, i.e. cardiovascular diseases, cancer, diabetes, neurodegenerative/-psychiatric diseases, musculoskeletal diseases, respiratory and infectious diseases, and their pre-clinical stages or functional health impairments. Across Germany, a random sample of the general population will be drawn by 18 regional study centres, including a total of 100,000 women and 100,000 men aged 20–69 years. The baseline assessments include an extensive interview and self-completion questionnaires, a wide range of medical examinations and the collection of various biomaterials. In a random subgroup of 20 % of the participants (n = 40,000) an intensified examination (“Level 2”) programme will be performed. In addition, in five of the 18 study centres a total of 30,000 study participants will take part in a magnetic resonance imaging examination programme, and all of these participants will also be offered the intensified Level 2 examinations. After 4–5 years, all participants will be invited for a re-assessment. Information about chronic disease endpoints will be collected through a combination of active follow-up (including questionnaires every 2–3 years) and record linkages. The GNC is planned for an overall duration of 25–30 years. It will provide a major, central resource for population-based epidemiology in Germany, and will help to identify new and tailored strategies for early detection, prediction, and primary prevention of major diseases.

## Introduction

Over the last 30 years, life expectancy has continuously improved, in part explained by a downwards trend in incidence rates of cardiovascular diseases and by improved clinical care for cardiovascular and other chronic diseases. Nonetheless, cardiovascular diseases and cancer, in particular, remain the by far most important causes of mortality, accounting for about 70 % of all deaths in Germany. Furthermore, the increases in overall life expectancy in Germany have been only partially matched by increases in expected life years free of disease-related disabilities [1]. Due to demographic changes the numbers of patients with stroke, chronic respiratory diseases, cancer, and neurologic or psychiatric disorders such as dementia and depression are increasing. Among the younger generations, an increasing prevalence of obesity is leading to growing numbers of patients with early-onset type-II diabetes mellitus [1]. Finally, chronic infections (e.g. viral hepatitis or human papillomavirus) and various diseases that may result from them, represent an often under-estimated burden in Germany and other industrially developed countries [1].

The abovementioned diseases constitute an increasing burden on the health care system, and also cause major losses in working capacity and related costs to the economy (cf. e.g. “Gesundheitsberichterstattung des Bundes” http://www.gbe-bund.de/). Health economic analyses suggest that disease prevention is a cost-effective way of improving general public health, even when medical costs in additional life years gained are taken into account [2, 3], and therefore prevention research remains an area of high priority for Germany. Effective strategies for the prevention of chronic diseases require accurate data about the causes of these diseases and about the potential magnitude by which chronic disease occurrences can be reduced by avoiding major risk factors.

Large, population-based prospective cohorts provide the ideal design for research on the combined effects of lifestyle, occupation and environment, social and psychosocial factors and genetic predisposition on disease development. A major challenge for such cohorts, however, is that they need to be very large, so as to allow for statistically powerful analyses on diverse disease outcomes. Given the high financial cost of a large-scale prospective cohort study as a national research infrastructure, it is of fundamental importance to plan and build the study from a multidisciplinary research perspective, and to allow a large number of medical and life science researchers from various research institutions, both nationally and internationally, to have access to the study resource.

The German National Cohort (GNC) builds on the experience of other medium-sized cohort studies like Cardiovascular Disease, Living and Ageing in Halle (CARLA), European Prospective Investigation into Cancer and Nutrition in Heidelberg und Potsdam (EPIC), Kooperative Gesundheitsforschung im Raum Augsburg (KORA), Heinz Nixdorf Recall Study (HNR, Risk Factors, Evaluation of Coronary Calcification, and Lifestyle in the Ruhr area) and Study of Health In Pomerania in Greifswald (SHIP), and the German National Health Examination Survey (GNIHES) [4–9]. Furthermore, the GNC study group collaborates with other large new cohorts, such as the UK-BIOBANK, CONSTANCES (France) or the LIFEGENE study [10–12] and major efforts are being made to harmonise instruments for data collection so as to allow future joint and comparative analyses.

### Study objectives

The GNC is designed to address research questions concerning a broad range of potential causes of major disease groups. The repeated collection of biomaterials in combination with extensive information from questionnaires and medical examinations is a basic asset of its design. The combination of these sources of information—repeated over time—will make it possible to specifically address pathways of disease development, providing clues to the biological mechanisms that may explain observed relationships. Overall, four general objectives are pursued by the National Cohort, and within their general scope a number of more specific research questions will be of particular focus.

#### Identification of etiological pathways from life-style and environmental risk factors to major chronic diseases and functional impairments

A first set of specific aims is to increase understanding of the role of particular risk factors in the development of major forms of chronic disease, with special emphasis on selected lifestyle factors (smoking, alcohol consumption, physical in-activity, nutrition, nutritional energy balance), as well as chronic infections, psychosocial factors, occupational and environmental influences and use of medications as possible causes. Using repeated measurements over time, it is aimed to obtain improved quantitative estimates for importance of major risk factors in terms of etiological and population-attributable fractions. In addition, and by integrating questionnaire data with various medical or functional examinations (Table 1) and analyses in bio-specimens, it is aimed to obtain novel insights into the natural history and causal pathways of disease development.

**Table 1:** Chronic diseases, pre-clinical phenotypes and functional measurements that will be ascertained in the German National Cohort

Chronic diseases	Intermediate phenotypes and measurements of function
CVD (myocardial infarction, heart failure, stroke, atrial fibrillation)	Subclinical atherosclerosis (arterial stiffness, ankle–brachial index, intima–media thickness of the carotid artery, MR imaging), cardiac dysfunction (electrocardiogram (ECG), 3D-echocardiography, MRI), elevated blood pressure
Diabetes mellitus	Impaired glucose tolerance (fasting glucose, oral glucose tolerance test), accumulation of advanced glycation end products in the skin (skin autofluorescence), retinopathy (retinal photographs)
Cancer	Precursor stages of haematologic malignancies (PBMCs)
Neurologic and psychiatric diseases (stroke, depression)	Mild cognitive impairment (cognitive functioning tests), olfactory function (smell test), brain MR imaging
Respiratory diseases (Chronic obstructive pulmonary disease, asthma)	Lung function (spirometry), airway inflammation (exhaled nitric oxide, FeNO), lung volume (MR imaging)
Infectious diseases	Seromarkers, immune function
Musculoskeletal diseases	Arthrosis, rheumatoid arthritis (Medical examination of knee, hip and hand joints)

#### Assessment of the geographic and socio-economic disparities in health status and disease risks in Germany and possible causes and explanations

A major objective of the GNC is to increase our knowledge about the fundamental causes for social and regional disparities in health. In addition to risk factors with a potential direct implication in disease aetiology from a biological, i.e. “mechanistic” perspective, special emphasis will also be given to socio-economic position (e.g., education, income, occupational status) and psychosocial factors (e.g., personality traits, chronic stress, workplace social environment) as health determinants. Furthermore, using secondary data sources (e.g. from health insurance records), information will be collected on the use of health services, specialised care, hospital care, medical interventions, and medication as important correlates of socio-economic inequalities in disease occurrence.

#### Improvement of risk prediction models for identifying individuals at increased risk of developing major chronic diseases, so as to allow personalised prevention strategies

Risk prediction models and algorithms for stratification of individuals into categories of lower and higher risk of developing major chronic diseases are important tools for personalised medicine and personalised prevention strategies [13–15]. The National Cohort will provide an outstanding resource for studies aiming to develop and/or validate comprehensive risk models that integrate risk factor information obtained by questionnaires, clinical examinations, whole-body magnetic resonance imaging (MRI), and the assessment of genetic and other biological markers in blood, urine and other bio-specimens (Table 3).

#### Evaluation of markers for early detection of diseases and pre-clinical phenotypes, as to develop effective tools for disease prevention

Detection of pre-clinical stages of diseases may increase cure rates, and/or help to minimize negative side-effects of treatment as well as help prevent the occurrence of incident disease. Stimulated by recent technological advances in the fields of genomics, transcriptomics, proteomics, metabolomics, epigenomics, and MRI, research on bio-markers of pre-clinical stages of disease is becoming a major theme for prevention [16–19]. The bio-specimen repository of the GNC will provide a valuable resource for the discovery and validation of novel biomarkers for early disease detection, at the same time ensuring a rapid connection between basic discovery research and its confirmation and validation in appropriately designed human population studies.

## Study design and methods

### Study population and recruitment

**Fig. 1:**
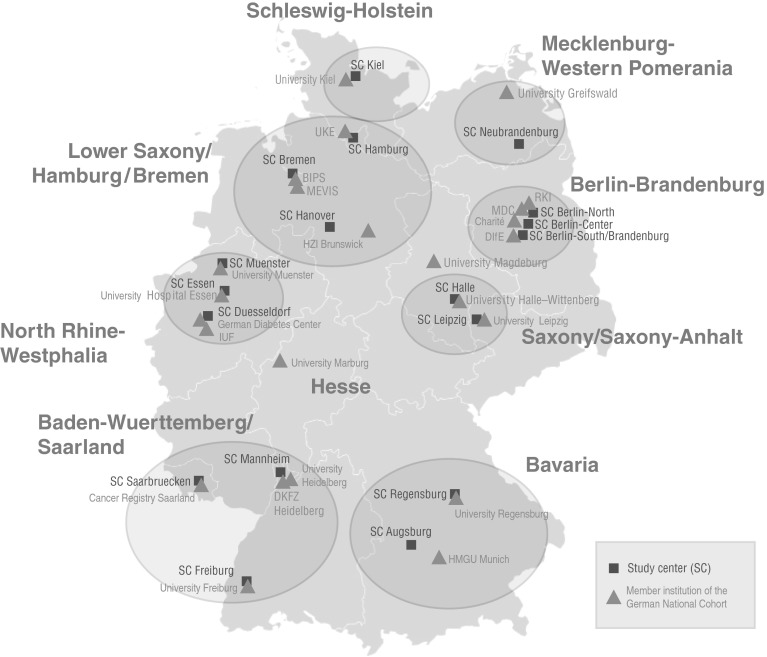
Study centres of the German National Cohort, grouped by regional cluster

The GNC will recruit a total of 200,000 residents in the age range of 20–69 years at baseline. Study participants will be recruited through a network of 18 study centres, covering mainly urban and industrialised areas and some rural regions throughout Germany. These centres are grouped into eight clusters working closely together (Fig. 1). Each centre will recruit a minimum of 10,000 cohort participants, drawn randomly from compulsory registries of residents in the study areas. The anticipated response rate is between 40 and 50 %. Currently there is no financial compensation for participation planned apart from reimbursement of travel costs. Participants will be invited to the local study centre where they will participate in a standardised, computer-assisted personal face-to-face interview (CAPI), complete self-administered questionnaires (partially on touch screen computers) and undergo a number of standardised physical and medical examinations. In addition, they will provide blood, urine samples and other biomaterials. All participants will receive a generalised letter of results in the weeks following their study visit. This letter will include some of the basic test results (e. a. blood test results, blood pressure data, anthropometric data, accelerometry data). For the MRI examinations, we have defined an exhaustive catalogue of chance findings that should either, or should not, be communicated to the study participants, to inform about potential health problems that would require further medical attention.

### Baseline examination and data collection

Data collection comprises two levels of intensity. All cohort participants will follow a 2.5 h recruitment protocol including the CAPI and questionnaire assessments and basic physical and medical examinations (*Level 1*), and a random sub-sample of 40,000 participants (20 % per study centre) will participate in an extended protocol that includes more in-depth physical and medical examinations (*Level 2*). In addition, at five MRI centres—in Augsburg, Berlin, Essen, Mannheim and Neubrandenburg—a total of 30,000 participants (drawn from the GNC participants of the respective study centres) will additionally undergo a high-resolution (3 T) MRI protocol for the acquisition of whole body, cardiac, and brain images, generating a comprehensive morphological and functional data base (*MRI programme*). The participants will either pertain to the random 20 % Level 2 sample or they will be offered the Level 2 examinations in addition to Level 1. The intensified sub-cohorts (Level 2 and MRI programme) will be used for more detailed studies on risk factors and early clinical precursor stages of diabetes, heart disease, and neurodegenerative disorders, in particular.

The baseline interview and self-administered questionnaires will cover socio-economic and socio-demographic factors, medical history, use of medications and health care, lifestyle factors, and questions related to environmental and occupational factors (Table 2). In addition, physical and medical examinations will be performed as specified in Table 3. Finally, all study participants will be asked to provide samples of blood, urine, saliva and stool, as well as nasal swabs (Table 3).

**Table 2:** Questionnaire data to be collected within the German National Cohort

Questionnaires
*Core questionnaire*
Socio-economic status (SES) and socio-demographic factors
Medical history
Family medical history
Medication of the last 7 days
Participation in screening programmes
Women’s/men’s questions
Smoking, alcohol, other drugs
Health-related and other quality of life (e.g. SF-12, IADL)
*Specific questionnaires*
Neurologic and psychiatric factors (symptom questionnaire, depression, anxiety disorders, headache, sleep) (MINI, PHQ-9, PHQ-7, RLS [RLS only Level 2])
Psychosocial factors (personality, chronic stress, childhood trauma, occupational stress, social network; conflicts at the work place and in the family, occupational insecurity) (BFI-10, PHQ stress, ERI, COPSOQ, childhood trauma)
Infections and immune function
Musculoskeletal diseases: Pain mannequin (only Level 2)
Oral health (OHIP-5)
Physical activity (at work, leisure time, and sport; activity patterns, setting)
Diet (FFQ, 24 h diet recall)
Environmental factors (postal addresses of work and home)
Occupation (screening questions for special exposures at work)
Health care utilization and compliance (GP, specialist consultation, hospital stays)

*Health*-*related and other quality of life* Health survey: SF-12; Instrumental Activities of Daily Living questionnaire: Amsterdam-IADL-Questionnaire. *Neurologic and psychiatric factors* Mini International Neuropsychiatric Interview: MINI; Patient Health Questionnaire: PHQ-7, PHQ-9; Cambridge-Hopkins RLS questionnaire: CHRLSq. *Psychosocial factors* The Big Five Inventory: BFI-10; Patient Health Questionnaire: PHQ-Stress; Effort-reward imbalance questionnaires: ERI; Copenhagen Psychosocial Questionnaire: COPSOQ. *Oral health* The Oral Health Impact Profile questionnaire: OHIP-5. *Diet* Food Frequency Questionnaire: FFQ. *Health care utilization and compliance* GP: Patient Survey

**Table 3:** Physical and medical examinations to be conducted within the German National Cohort at baseline recruitment, by study level (Level 2 exams in addition to Level 1 exams)

Study element	1st visit	
	Level 1	Level 2
*Cardiovascular system*		
Blood pressure and heart rate	x	x
Electrocardiography (10-s ECG; 12 leads)	x	x
3D Echocardiography	x	x
Ankle–brachial index (ABI), measures of arterial stiffness (pulse wave)	x	x
Long-term ECG/apnea device		x
*Diabetes*		
Oral glucose tolerance test (OGTT; n = 40,000 including Level 1 + 2 participants)	x	
AGE reader (skin autofluorescence)		x
*Cognitive function*		
Semantic memory, episodic memory, working memory, attention/executive, motor coordination	x	x
Numerical reasoning (fluid intelligence), passive vocabulary (crystallized intelligence)		x
*Lung function*		
Spirometry	x	x
Nitric oxide (FeNO) in exhaled air		x
*Musculoskeletal system*		
Medical examination of knee, hip and hand joints for arthrosis and rheumatoid arthritis		x
*Oral health*		
Tooth count	x	x
Medical examination of the oral cavity for periodontal disease and temporomandibular disorders		x
*Sensory organs*		
Ophthalmological measurements		
(retinal photography, simple visus test)		x
Hearing test: speech in noise number triple test (application via touch screen and headphone)		x
Olfactory test (Sniffin’ sticks 12)	x	x
*Physical activity, physical fitness*		
7-Day accelerometry	x	x
Submaximal bicycle ergometry		x
Hand grip strength	x	x
*Anthropometry*		
Body weight and body height (standing)	x	x
Waist and hip circumference	x	
Body impedance analysis (BIA)	x	
Ultrasound of the abdominal fat distribution	x	
*Bio*-*specimens*		
Serum	6 ml (30 aliquots)	
Plasma	9,6 ml (48 aliquots)	
Erythrocytes	1,2 ml (6 aliquots)	
Lymphocytes for DNA	2.4 ml (4 aliquots)	
RNA tubes	1	
Urine^a^	6 ml (12 aliquots) + 2,4 ml (4 aliquots)	
Saliva	1.2 ml (2 aliquots)	
Nasal swab	2 swabs per tube	
Stool	1 tube with DNA stabilisation reagent (8 ml)	

^a^Spot urine will be collected during the time the participants are at the study centre

### Repeat data collections and medical examinations over time

All participants of the GNC will be re-invited for a second examination (re-assessment) 4–5 years after their baseline recruitment which will include about the same examinations (except long-term ECG and oral glucose tolerance test) as at baseline at Level 1 and 2, but with a reduction in the number of aliquots collected for the bio-specimens. For the MRI programme, funding has to be sought for the repeat-examination as the current funding only covers the baseline MRI examination. At re-assessment, intra-individual, medium-term changes in risk factors and prospective changes in quantifiable preclinical morbidity characteristics and incident clinical disease can be investigated. For the re-assessment, we anticipate a re-participation rate of at least 75 % of all cohort participants. This estimate is based on experiences from a number of ongoing studies in Germany, including KORA (Augsburg), HNR (Essen), SHIP (Greifswald), CARLA (Halle) and the German National Health Interview and Examination Survey 1998 (GNHIES)/DEGS survey for adults [5–7, 9, 20, 21].

In addition to the re-assessment after 4–5 years, short-term (0.5-year) replication studies will be embedded in the cohort, using a 6,000-subject sub-sample of participants of the first (baseline) visit, and a 4,000-subject subsample of participants in the second visit, proportionally spread over all 18 study centres. These reliability studies will serve to estimate within-subject “random” variations in risk factor measurements, so as to allow corrections of attenuation effects resulting from random misclassification.

### Prospective follow up for vital status and case ascertainment of incident diseases

As from their first enrolment into the GNC, all cohort participants will be re-contacted every 2–3 years and asked to fill in short questionnaires about changes in lifestyle and other characteristics (e.g., use of medications, smoking, menopausal status, selected disease symptoms) and about the occurrence of physician-diagnosed diseases or of triggering events for selected major diseases, such as visiting a cardiologist or having been hospitalised (“active” follow-up). Additional telephone contacts and interviews will be used for participants who do not respond to written invitations. Self-reported cases of novel onset major diseases will be verified systematically by contacting the participant’s pertinent physician(s) and/or hospitals at which they were treated, and coded according to the International Classification of Diseases (ICD) [22]. The list of diseases for which this form of follow-up will be the principal method of prospective case ascertainment includes coronary heart disease/myocardial infarction, heart failure, atrial fibrillation, type-2 diabetes mellitus, cerebrovascular disease (stroke), depression, cancer, and chronic infectious diseases. With regard to cancer occurrences, follow-up will also be based on systematic record linkages with existing epidemiological cancer registries (“passive” follow-up), which are mandatory now and soon will cover all regions of Germany. For other morbidities, it is planned to complete self-reports by data obtained through linkage to health insurance records plus systematic queries at large clinics and hospitals.

With regard to vital status and mortality, follow-up will be performed through queries to registries of residents and authorities (“Einwohnermeldeämter”, “statistische Landesämter”, “Gesundheitsämter”). While the latter are common practice in many epidemiological studies, they are relatively labour-intensive. A new German National Death Index (NDI), which is presently being planned (independently of the GNC), will make the follow-up for mortality more efficient in future years.

### Linkage to secondary data to obtain additional information on the individual’s health and employment history

#### Data from statutory health insurances

About 85 % of the population in Germany is insured by the statutory health insurance system, and the GNC is setting up cooperation structures with data holding institutions to allow individual data linkage for participants. The health insurance data contain information about disease diagnoses and treatment, ambulatory and hospital care and prescribed medications. The major objective of the linkage of health insurance data is to obtain information on the use of health services and medications, for health services research and research on medications and interventions as possible determinants of disease outcomes and prognosis. In addition, however, the information may be used as an additional source of information on disease incidence.

#### Data on occupational history

More than 95 % of the German workforces are member of the compulsory statutory pension insurance system. These insurances hold socio-demographic data of the employees, as occupational code, and job position, provided by the employer on an at least annual basis. The legal entity that holds all these data and is allowed (within strict limits) to use it for research purposes is the Institute for Employment Research (IAB). The GNC will cooperate with IAB to augment the data of the participants with respect to occupational history.

#### Geocoded environmental data

Data on environmental exposures will be generated by the combination of occupational and home addresses with available geocoded exposure data, e.g., for air pollution, background radiation, noise and heat.

### Time line

**Fig. 2:**
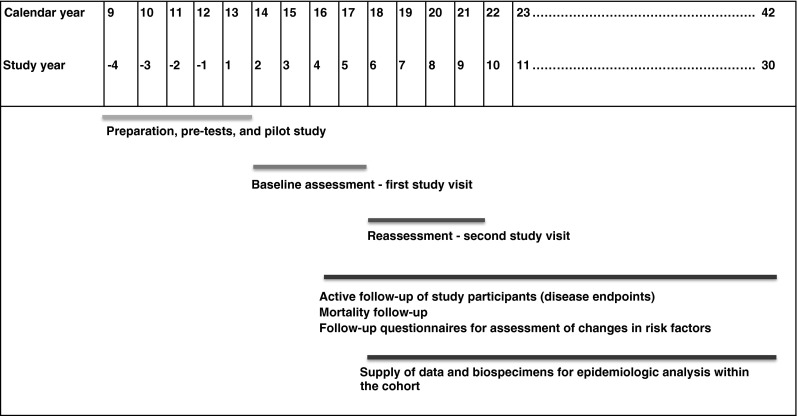
Roll-out of the German National Cohort—overall timeline

The projected time frame for the National Cohort covers a period of 25–30 years (Fig. 2). The recruitment will start with a pilot phase in the 2nd half of 2013. Start of full-scale recruitment is planned for the beginning of 2014. Shortly after completion of recruitment, the study centres will start with the re-assessment of the entire cohort.

### Expected case numbers for major chronic diseases, and statistical power considerations

For a project that has the size and scope of the GNC, developing a notion of necessary study size is a complex exercise. Besides the anticipated statistical evaluation for a great variety of possible study questions, the total number of study participants planned was also carefully balanced against the depth of phenotyping and detail of data collected from the study participants. One basic guideline was that, with regard to a number of chronic endpoints such as major forms of cancer and cardiovascular diseases, the cohort study should be large enough to allow future, stand-alone evaluations within the GNC for basic risk factor associations. For the most frequent diseases, such as diabetes or myocardial infarction, the GNC should also allow the development of detailed, multivariable risk models including large numbers of potential risk determinants. For rarer forms of disease or pre-clinical phenotypes the GNC should be large enough to make a meaningful contribution to international cohort consortia.

Table 4 presents the expected case numbers for the most frequent forms of cancer and for selected non-cancer chronic endpoints. Standard calculations indicate that studies which include at least 300–500 cases of a specific disease, with control-to-case ratios varying between 2:1 and 4:1, will have a statistical power of 0.80 (at a significance level of 0.05) to detect an odds ratio of about 1.4–1.6 for a binary exposure with 20 % population prevalence, or an odds ratio of about 1.5–1.7 for top versus bottom quartile categories of an exposure or risk factor. The number of 300–500 incident cases of disease corresponds, for example, to the rarer forms of cancer listed in Table 4 or to incident cases of type 2 diabetes among adults <40 years of age in the “Level 2” sub-cohort with intensified phenotyping. With 1,000–2,500 cases, as obtained for more frequent diseases at both study levels after 10 years, a statistical power (at significance level 0.05) of 0.80 or higher is given to detect exposure-disease associations corresponding to an expected odds ratio of around 1.15–1.3 between the extreme quartiles. 1,000–2,500 incident cases are expected to be observed for the more frequent cancer types, and in the intensified sub-cohort (N = 40,000) similar case numbers are also expected for diabetes and common CVD endpoints. At still higher levels of incidence, with 5,000 events and more (e.g., diabetes, frequent forms of cardiovascular disease and overall mortality), the minimally detectable odds ratios are even lower, and studies will have high statistical power also for complex forms of statistical modelling, e.g. involving interaction terms with rare exposures (e.g. genetic variants).

**Table 4:** Expected counts of incident cancer cases and incident non-cancer cases after 5, 10, 15 or 20 years of average follow-up, for the overall cohort (N = 200,000) or for the intensified sub-cohort (N ~ 60,000)

Disease	Expected cumulative incidence at study Level 1 (200,000 subjects)				Expected cumulative incidence at study Level 2 (~60,000 subjects^a^)			
	Average follow-up duration (years) (+corresponding calendar date)							
	5 years (2022)	10 years (2027)	15 years (2032)	20 years (2037)	5 years (2022)	10 years (2027)	15 years (2032)	20 years (2037)
Any cancer	5,100	13,000	21,000	29,000	1,500	3,900	6,000	9,000
Breast	780	1,800	2,900	4,000	240	555	885	1,200
Prostate	720	1,900	3,200	4,600	210	570	960	1,365
Colon, Rectum	670	1,800	3,100	4,500	195	540	930	1,335
Lung	560	1,400	2,400	3,400	165	435	720	1,020
Bladder	260	710	1,200	1,800	75	210	375	540
Kidney	190	500	850	1,200	60	150	255	360
non-Hodgkin L.	140	340	580	820	45	105	180	240
Pancreas	120	330	580	830	30	105	180	255
Corpus Uteri	120	320	540	770	30	90	165	300
Brain + CNS	90	200	330	450	30	60	105	135
Ovary	110	260	440	610	30	75	135	180
Myocardial infarction	1,700	4,400	7,300	10,000	525	1,305	2,250	3,150
Stroke	1,600	4,300	7,500	11,000	465	1,290	2,250	3,300
Diabetes	5,800	13,000	21,000	28,000	1,800	4,050	6,300	8,400
Rheum. arthritis	250	590	940	1,300	75	180	285	375
COPD	2,300	5,800	9,700	13,000	690	1,740	2,850	4,050
Heart failure	1,600	4,600	8,200	12,000	480	1,380	2,550	3,600
Mortality	4,600	14,000	26,000	47,000	1,365	4,050	7,950	13,950

Calculations based on age-specific incidence from German Cancer Registries [23]

^a^40,000 participants in the representative Level 2 study programme plus ~20,000 participants in the MRI study, who will additionally also have the Level 2 examinations

For studies of rarer disease outcomes, as well as studies that require extremely large numbers of endpoints (e.g., for comprehensive modelling of gene–environment interactions involving genetic variants with low allele frequency) the GNC consortium will collaborate with other large-scale cohorts in Europe, such as UK-Biobank, Constances, or the LifeGene study [10–12].

## Study organization

### Central data management

At all recruitment centres data will be collected through web-based, standardised data entry forms and protocols for interviews and questionnaires as well as for all physical and medical examinations. The data are directly integrated in two data integration centres, at the University of Greifswald and at the German Cancer Research Center (DKFZ), which will share the task of offering data collection services (including constant on-line support) to the 18 study centres. The 2 integration centres offer identical services, each to half of the 18 study centres (divided geographically into a northern and a southern region), and will ensure continuous mutual back-ups of the full datasets collected from all 18 centres. Also, at any time both integration centres will be able to take over each other’s data collection services, covering the other half of the study centres as a back-up service.

Prospective record linkage, a master participant index, and prospective consent management will be performed by a trusted third party separate from the main study data base. The trusted third party maintains all linkages to external secondary data sources and conducts prospective vital status follow-up.

### MRI programme

For the MRI programme, five dedicated GNC MR imaging centres will be established, with the instalment of new 3-T MRI devices. Identical equipment is being installed at all these centres, so as to allow the highest possible degree of standardization of the whole-body MRI examinations in terms of image quality and internal validity, and for reasons of cost-effectiveness. MRI technologists from each study site receive centralised training and certification by a central imaging core. In total, 30,000 subjects will be examined by MRI, and all subjects undergoing the MRI programme will also participate in the Level 2 examinations.

To deal with incidental findings, a group of MRI specialists is currently preparing a list of life-threatening observations and a final Standard Operating Procedures (SOP) for their communication. Only these will be communicated to the participants.

### Collection and storage of bio-specimens

From all cohort participants various bio-specimens will be collected, i.e., whole blood, serum, EDTA plasma, erythrocytes, RNA, urine, saliva, nasal swabs, stool. To guarantee the highest quality of the samples, the collection and processing of the samples will be highly standardised and includes the use of an automated liquid handling system for sample aliquoting. In addition, a detailed and comprehensive bio banking concept was elaborated. Two-thirds of each individual’s aliquots collected during baseline recruitment and the subsequent re-assessment will be stored in a centralised and fully automated bio-repository, which will be established at the German Centre for Environmental Health (HMGU Munich). One-third of each individual’s biological specimens will be stored at the local study centres for use in local analyses and as back-up storage.

### Quality management (QM)

To guarantee a high quality of data collection, processing and storage throughout the GNC, a comprehensive QM system will be established, including central structures for internal and external QM. The Robert Koch Institute (RKI) will be responsible for the external QM, while the internal QM will be centrally organised by a QM officer based in the central executive office of the GNC and will conducted by the study centres themselves. For data collection and medical examinations standardised instruments will be applied, and detailed SOPs will be used in all centres. The study personnel will undergo an extensive training and will be centrally certified for the complete data collection including all examinations, application of questionnaires and recruitment procedures. Repeated centralised training and recertification will take place at regular intervals.

### Ethics and data confidentiality

The GNC will be performed in compliance with the Federal Data Protection Acts [24] and all other pertinent legislation and directives. Furthermore, the ‘Opinion of the German Ethics Council’ [25] on human bio-banks for research as well as other ethically relevant directives will be observed. The aim is to secure trust into the way data and bio-samples are stored and handled. A data protection concept for the GNC was developed in close collaboration with the German federal and state commissioners for data protection and freedom of information. An external ethics advisory board has been established which will accompany the GNC over the full study period. A ‘Code of Ethics’ of the GNC (“Ethikkodex”) has been developed, describing general rules and principles for ethical collection and use of study data (www.nationale-kohorte.de), and the study will be under steady surveillance by the ethical committees of the regional study centres. Special emphasis will be placed on privacy, safety of data, especially genetic information and reporting of incidental medical findings.

## Funding, governance and management

As a long-term project and scientific infrastructure, the GNC needs to secure long-term funding. For the first 10 years, which will cover baseline assessment and re-assessment of all 200,000 study participants, as well as a first phase of prospective follow-up for disease occurrence, the German federal and local state governments and the Helmholtz Association cover the main part of the overall budget, and approved a budget plan for the first 10 years of the project. This will be complemented by considerable in-kind contributions of all participating institutions.

**Fig. 3:**
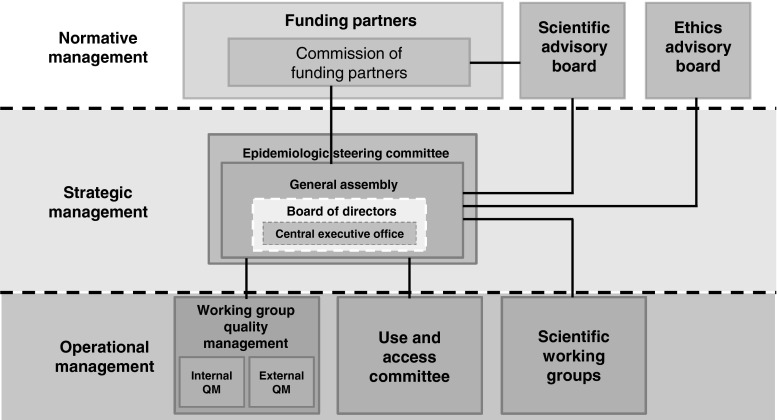
Scheme of governance and management structures of the German National Cohort

As a long-term scientific project, with participation of a large number of institutions, the GNC requires special governance and management structures. The central governing body is the formally established GNC e.V. as a registered association (since February 2013) in which all participating research institutions are members. The general assembly of the association has elected a board of directors as who will be responsible for the implementation of the government and management of the GNC, including four scientific directors and one administrative director. A commission of funders acts as a supervisory board regarding general governance questions and project decisions with possible financial impact (Fig. 3).

The society’s general assembly has formed several committees and working groups, including an *epidemiological steering committee* (composed of 1–2 scientists per participating scientific institution), and a working group for *Quality Management*. A number of *scientific and thematic working groups*, involving many scientists across Germany, have been very active in developing the GNC study protocol, and it is planned that these will be continued as platforms for the planning of scientific use of the GNC data and bio-specimens. In addition to these internal organization structures, the GNC receives external guidance by a *Scientific Advisory Board* and an *Ethical Advisory Board*.

With regard to the *rights of access* to and use of study data and bio-specimens explicit and detailed rules have been defined, which can be found on the homepage of the GNC (http://www.nationale-kohorte.de/nutzungsordnung.html). A use and access committee will review all applications for the use of data and bio samples of the GNC and suggest decisions to the General Assembly and the Board of Directors.

## Conclusion

The GNC will provide a central platform for future epidemiological research in Germany, with a strong potential to push the development of new strategies for prevention, early detection and prognosis of major chronic diseases. With its broad spectrum of examinations and the systematic re-assessments of all study participants, and its broad spectrum of high-quality biomaterials a cohort of this size provides an excellent tool for future, population-based longitudinal research. The large-scale, embedded MRI programme is another unique asset of the GNC. One special element of the GNC is the fact that virtually the whole German epidemiological community has collaborated in the design and preparation of the GNC, and many of them will be directly involved in the field work. The GNC thus constitutes an extraordinary basis for scientific cooperation and networking amongst epidemiologists and other health scientists in Germany.
